# Effect of *Saccharomyces cerevisiae* culture mitigates heat stress-related dame in dairy cows by multi-omics

**DOI:** 10.3389/fmicb.2022.935004

**Published:** 2022-07-15

**Authors:** Dewei Du, Wenbo Jiang, Lei Feng, Yu Zhang, Peng Chen, Chengqiang Wang, Zhiyong Hu

**Affiliations:** ^1^Ruminant Nutrition and Physiology Laboratory, College of Animal Science and Technology, Shandong Agricultural University, Tai’an, China; ^2^Beijing Enhalor International Tech Co., Ltd., Beijing, China; ^3^College of Life Sciences, Shandong Agricultural University, Tai’an, China

**Keywords:** heat stress, dairy cows, *Saccharomyces cerevisiae* culture, metagenomics, metabolomics

## Abstract

The effect of heat stress on ruminants is an important issue. In recent years, the growth of the Chinese dairy industry has rapidly increased, generating RMB 468,738 million revenue in 2021. A decreased milk yield is the most recognized impact of heat stress on dairy cows and results in significant economic loss to dairy producers. Heat stress also lowers immunity and antioxidant capacity and changes the bacterial composition and metabolites of the rumen. The purpose of this study was to investigate the effect of addition *Saccharomyces cerevisiae* culture on heat-stressed cows. The impact of *S. cerevisiae* culture on microbiota composition, functional profiles, and metabolomics was assessed in heat-stressed cows. A total of 45 Holstein cows in mid-lactation were selected and randomly divided into three groups (15 cows per group). Groups D-C, D-A, and D-B were fed with the basal diet, the basal diet + first *S. cerevisiae* culture 100 g/day, and the basal diet + second *S. cerevisiae* culture 30 g/day, respectively. The trial lasted 60 days. There was an increased abundance of the Phylum Firmicutes in the rumen of heat-stressed dairy cows fed with *S. cerevisiae*, of which four genera had significantly higher abundance, *Ruminococcus_gauvreauii_group, Butyrivibrio_2, Moryella*, and *Ruminiclostridium_6.* At the functional level, ten pathways differed significantly between the three groups (*P* < 0.05), with an increase in fatty acid biosynthesis, fatty acid metabolism, PPAR signaling pathway, ferroptosis, and biotin metabolism in the treatment groups. More differential metabolites were found in the D-C and D-A groups than in the D-C and D-B groups. These results indicate that *S. cerevisiae* cultures can influence the health status of heat-stressed cows by modulating rumen microbial composition, function, and metabolites, thereby improving rumen cellulolytic capacity. This study can provide or offer suggestions or recommendations for the development and utilization of feed additives.

## Introduction

According to the United Nations Intergovernmental Panel on Climate Change (IPCC) report, the burning of fossil fuels and deforestation (Gray, 2007; Jianbin, 2013) accelerate the pace of global warming, which will be a big challenge for the dairy industry in China (Zhang et al., 2020), impacting the success of its dairy industry. Shandong Province in East China has a temperate monsoon climate with high summer temperatures, and cows in this region are severely affected by heat stress. This has led to a decrease in milk production and feed intake and an increase in the incidence of intramammary bacterial infection (IMI) (West, 2003; Rakib et al., 2020). Heat stress also lowers immunity and antioxidant capacity (Tao et al., 2012; Du et al., 2022) and changes the bacterial composition and metabolites of the rumen (Zhao et al., 2019). This is detrimental to the health of cows and causes serious losses to the dairy industry.

The gastrointestinal tract of cows is a complex ecosystem that has a strong impact on health (Zeineldin et al., 2018a). The rumen, the first stomach of ruminants, has a complex and diverse microbiota that can digest cellulose and provide nutrients to the organism (Bickhart and Weimer, 2018). The rumen microbiota can also stimulate and improve immune responses (Zhang et al., 2021). Recent studies have shown that the negative effects of heat stress on cows can be ameliorated by adding probiotics or other additives to the feed (Liu et al., 2014; Ye et al., 2016; Shah et al., 2020; Li et al., 2021a). These studies have shown varying degrees of improvement in milk production, immunity, and antioxidant capacity, as well as changes in the composition of the rumen microbiota and metabolites following the addition of these products. This may be due to the critical role played by host-rumen-microbiota interactions, and these additives alter the composition of the rumen flora and its microbial metabolites, thereby improving the health of the cow. The same argument is made in a review by Zeineldin et al. (2018b).

Our previous studies showed that *Saccharomyces cerevisiae* cultures could improve the performance, antioxidant capacity, and immunity of heat-stressed cows and reduce corporate losses (Du et al., 2022). Previous studies on heat-stressed cows have centered on rumen microbial diversity and metabolomics, and this experiment instead focused on rumen microbial diversity, metagenomics, and metabolomics. The objective of this study was to determine the effect of the addition of *S. cerevisiae* cultures on rumen microbial composition, rumen microorganisms’ function, and rumen metabolites.

## Materials and methods

### Animals, diets, and trial design

This study was conducted at the cattle farm of Xianghe Dairy Co., in Zaozhuang City, Shandong Province. A total of 45 Holstein cows in mid-lactation with a similar milk yield (29.4 ± 3.7), parity (1.8 ± 0.6), days in lactation (158 ± 14), and health were selected and divided into three groups of 15 cows each in a randomized complete block design. The control group (D-C) was fed with a basal diet, while the treatment groups were fed with a basal diet + the first *S. cerevisiae culture* at a rate of 100 g/day (D-A) or a basal diet + the second *S. cerevisiae* culture at a rate of 30 g/day (D-B). Both *S. cerevisiae* cultures were provided by Beijing Enhalor International Tech Co., Ltd. Cows in the treatment groups were fed with *S. cerevisiae* culture separately during the morning feeding. The trial lasted for 60 days, with 10-day and 50-day adaptation and formal trial periods, respectively. The average temperature of the dairy barn during the formal trial period was a minimum of 26.9°C. Cows were under heat stress at all times during the trial.

### Rumen fluid sampling

Two hours after feeding the cows in the morning of day 50 of the formal trial period, the rumen fluid from 45 cows was sampled using a portable rumen fluid sampling tube. Rumen fluid (100 ml) was collected per cow, filtered through four layers of gauze, packed into 2 ml freezing tubes, and preserved in liquid nitrogen.

### DNA extraction and sequencing

DNA was extracted from ruminal fluid samples using the HiPure Stool DNA Kits (Magen, Guangzhou, China) according to the manufacturer’s protocols. NanoDrop2000 was used to determine the purity of the extracted DNA. The 16S rDNA V3-V4 region was amplified by PCR using the following primers: 341F, 5′-CCTACGGGNGGCWGCAG-3′, and 806R 5′-GGACTACHVGGGTATCTAAT-3′. Amplicons were extracted from 2% agarose gels, purified using the AxyPrep DNA Gel Extraction Kit (Axygen Biosciences, Union City, CA, United States) according to the manufacturer’s instructions, and quantified using an ABI StepOnePlus Real-Time PCR System (Life Technologies, Foster City, CA, United States). Purified amplicons were pooled in equimolar ratios and paired-end sequenced on an Illumina platform (PE250) according to standard protocols.

### Bioinformatics analysis

Raw reads were further filtered using FASTP to obtain high-quality clean reads (Chen et al., 2018a) (version 0.18.0). Paired-end clean reads were merged as raw tags using FLASH (Magoč and Salzberg, 2011) (version 1.2.11) with a minimum overlap of 10 bp and mismatch error rates of 2%. Noisy raw tag sequences were filtered under specific filtering conditions (Bokulich et al., 2013) (QIIME version 1.9.1) to obtain high-quality clean tags. The filtering conditions are as follows: (1) break raw tags from the first low-quality base site where the number of bases in the continuous low-quality value (the default quality threshold is ≤3) reaches the set length (the default length is 3 bp); (2) then, filter tags whose continuous high-quality base length is less than 75% of the tag length. The clean tags were clustered into operational taxonomic units (OTUs) with *h* ≥ 97% similarity using the UPARSE (Edgar, 2013) (version 9.2.64) pipeline. All chimeric tags were removed using the UCHIME algorithm (Edgar et al., 2011), and effective tags were obtained for further analysis.

The representative OTU sequences were classified into organisms using a naive Bayesian model with the RDP classifier (Wang et al., 2007) (version 2.2) based on the SILVA database (version 132), with a confidence threshold value of 0.8. Chao1, ACE, Shannon, and Simpson were calculated using QIIME (Caporaso et al., 2010) (version 1.9.1) and plotted histograms using the omicsmart online tool.^1^ Principal coordinate analysis (PCoA) of unweighted-unifrac distances was generated using the R Vegan package (Oksanen et al., 2012) (version 2.5.3) and plotted using the R ggplot2 package (Wickham, 2011) (version 2.2.1). The stacked bar plot of the phylum and genus composition was visualized using the R ggplot2 package (version 2.2.1). The GraphPad Prism version 8.0.2 software was used to draw horizontal bar charts.

### DNA extraction, metagenomic sequencing, and metagenomic data processing

Genomic DNA was extracted using HiPure Bacterial DNA Kits (Magen, Guangzhou, China) according to the manufacturer’s instructions. DNA quality was detected using Qubit (Thermo Fisher Scientific, Waltham, MA, United States) and Nanodrop (Thermo Fisher Scientific, Waltham, MA, United States). Qualified genomic DNA was first fragmented by sonication to a size of 350 bp and then end-repaired, A-tailed, and adaptor-ligated using the NEBNext^®^ MLtra™ DNA Library Prep Kit for Illumina (NEB, Ipswich, MA, United States) according to the preparation protocol. DNA fragments with a 300–400 bp length were enriched by PCR, PCR products were purified using the AMPure XP system (Beckman Coulter, Brea, CA, United States), and libraries were analyzed for size distribution using the 2100 Bioanalyzer (Agilent, Santa Clara, CA, United States). Genome sequencing was performed on the Illumina NovaSeq 6000 sequencer using pair-end technology (PE 150).

Raw data from the Illumina platform were filtered using FASTP (version 0.18.0) (Chen et al., 2018b) by removing reads with any of the following: (1) ≥10% unidentified nucleotides (N), (2) ≥50% bases having Phred quality scores ≤20, or (3) aligned to the barcode adapter. After filtering, clean reads of each sample were assembled individually using MEGAHIT (version 1.1.2) (Li et al., 2014). Genes were predicted based on the final assembly contigs (>500 bp) using MetaGeneMark (version 3.38) (Zhu et al., 2010). The predicted genes ≥300 bp in length from all samples were pooled and combined based on ≥95% identity and 90% read coverage using CD-HIT (version 4.6) (Fu et al., 2012) to reduce the number of redundant genes for the downstream assembly step. The reads were realigned to the predicted gene using Bowtie (version 2.2.5) (Langmead and Salzberg, 2012) to count the read numbers. The final gene catalog was obtained from non-redundant genes with gene reads > 2. Quantitative analysis of genes was conducted using Bowtie (version 2.2.5) and PathoScope (version 2.0.7). The unigenes were annotated using DIAMOND (version 0.9.24) (Buchfink et al., 2015) by aligning them with the Kyoto Encyclopedia of Genes and Genomes (KEGG). The histogram of the difference was drawn using the omicshare online tool (see text footnote 1). The Kruskal-Wallis test was adopted. Meanwhile, Tukey’s HSD test was used to understand where the differences originated.

### Analysis of the rumen metabolome

The samples (1 mL) were freeze-dried and resuspended in 100 μL prechilled 80% methanol by well vortex, incubated on ice for 5 min, and centrifuged at 15,000 *g* at 4°C for 15 min. The supernatants were diluted to a final concentration containing 53% methanol using LCMS-grade water. The samples were subsequently transferred to fresh Eppendorf tubes and centrifuged at 15,000 *g* at 4°C for 15 min. Finally, the supernatants were injected into the LCMS/MS system for analysis.

UHPLC-MS/MS analyses were performed using a Vanquish UHPLC system (Thermo Fisher, Germany) coupled with an Orbitrap Q ExactiveTM HF-X mass spectrometer (Thermo Fisher, Germany) from Gene *Denovo* Co., Ltd. (Guangzhou, China). Samples were injected onto a Hypersil GOLD column (100 × 2.1 mm, 1.9 μm) using a 17-min linear gradient at a flow rate of 0.2 ml/min. The eluents for the positive polarity mode were eluent A (0.1% FA in Water) and eluent B (Methanol), and the eluents for the negative polarity mode were eluent A (5 mM ammonium acetate, pH 9.0) and eluent B (Methanol). The solvent gradient was set up as follows: 2% B, 1.5 min; 2–100% B, 12.0 min; 100% B, 14.0 min; 100–2% B, 14.1 min; 2% B, 17 min. A Q ExactiveTM HF-X mass spectrometer was operated in the positive/negative polarity mode with a spray voltage of 3.2 kV, capillary temperature of 320°C, sheath gas flow rate of 40 arb, and aux gas flow rate of 10 arb.

The raw data files generated by UHPLC-MS/MS were processed using the Compound Discoverer 3.1 (CD3.1, Thermo Fisher) to perform the peak alignment, peak picking, and quantitation of each metabolite. The main parameters included are as follows: retention time tolerance, 0.2 min; actual mass tolerance, 5 ppm; signal intensity tolerance, 30%; signal/noise ratio, 3; minimum intensity, 100,000. The peak intensities were normalized to the total spectral intensity. The normalized data were used to predict the molecular formula based on additive ions, molecular ion peaks, and fragment ions. The peaks were then matched with the mzCloud,^2^ mz Vaultand Mass List database, to obtain accurate qualitative and relative quantitative results. Statistical analyses were performed using the statistical software, namely, R (R version R-3.4.3), Python (Python 2.7.6 version), using CentOS to run this software (CentOS release 6.6). When data were not normally distributed, normal transformations were attempted using the MaxQuant (MaxQuant version 1.6.17.0). The abundance of differential metabolites in the same group was normalized using the *z*-score, and the VIP score of OPLS-DA was used to draw the graph. The omicsmart online tool was used to draw differential KEGG enrichment bubble map and VIP score.^3^

## Results

### 16S rDNA sequencing analysis

16S rDNA in the rumen fluid sample was filtered to obtain clean reads (758,403 ± 3,568 reads in the D-C group, 772,109 ± 4,155 reads in the D-A group, and 760,383 ± 3,892 reads in the D-B group). Our study also obtained 25,312 OTUs.

### Bacterial diversity analysis

**FIGURE 1:**
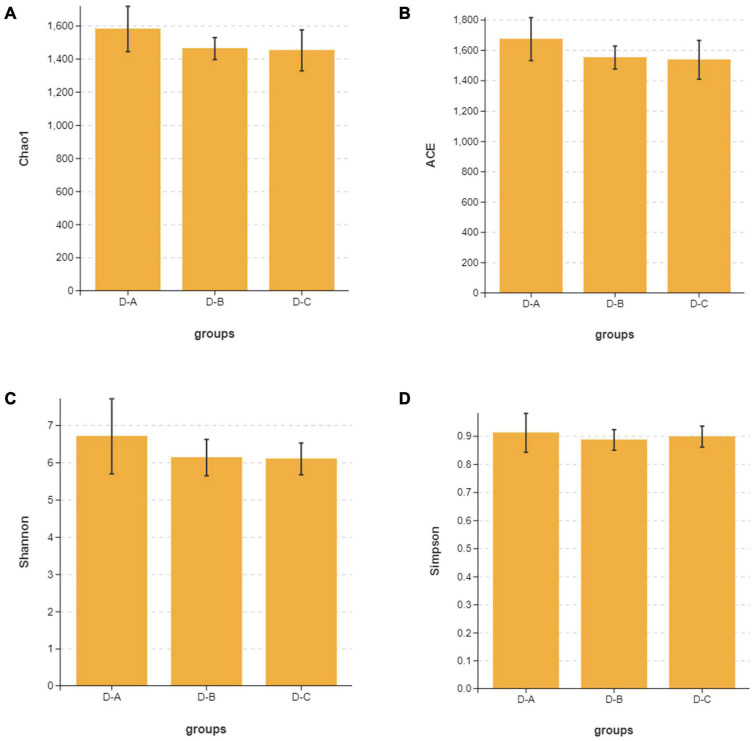
The **(A)** Chao1, **(B)** ACE, **(C)** Shannon, and **(D)** Simpson alpha diversity indices for the rumen microbiota.

**FIGURE 2:**
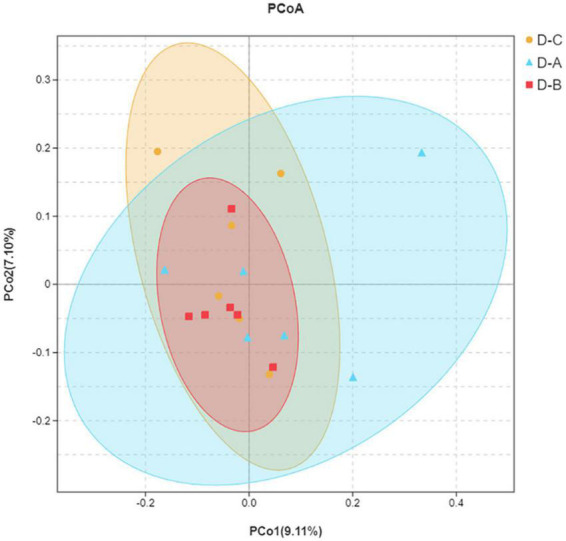
Principal coordinate analysis (PCoA) plot. The closer distance between the sample points indicates a higher similarity between the bacteria.

Alpha diversity indices, including Chao1, ACE, Shannon, and Simpson, showed that microbial community diversity in the rumen fluid samples was higher in the D-A group than in the other two groups (Figures 1A–D); however, this difference was not significant (*P* > 0.05). Beta diversity showed that there was no significant separation between the three groups of microbiota, indicating that there were no significant differences between them (Figure 2). There was also no significant separation at the OTU level using the ANOSIM test with R2 = –0.0095 (*P* = 0.549) (Supplementary Figure 1).

### Rumen microbial composition at the phylum and genus levels

**FIGURE 3:**
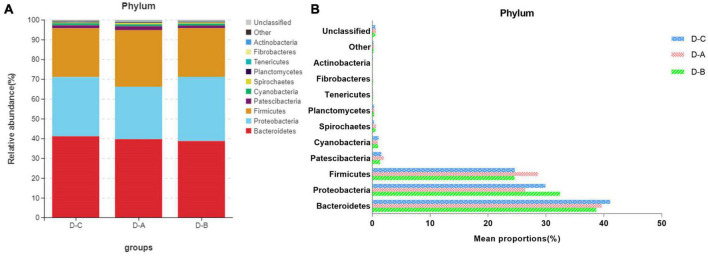
Relative rumen microbiota abundance at the **(A,B)** phylum level in the D-C, D-A, and D-B groups.

**FIGURE 4:**
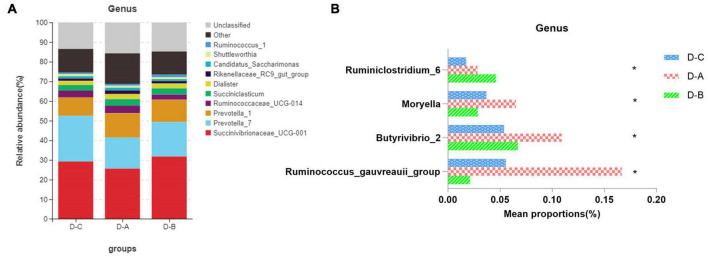
Relative rumen microbiota abundance at the **(A,B)** genus level in the D-C, D-A, and D-B groups. *P < 0.05.

Figure 3 shows the top ten most abundant microbial species in the three groups. Of these phyla, *Bacteroidetes*, *Proteobacteria*, and *Firmicutes* had the highest abundances of 39.8 ± 2.02, 29.6 ± 3.7, and 25.9 ± 1.7%, respectively. The amount of *Firmicutes* was higher in the D-A group than in the other two groups, while the abundance of the *Proteobacteria* was lower in the D-A group, but these differences were not statistically significant (*P* > 0.05). The dominant genera were *Succinivibrionaceae_UCG-001* (28.9 ± 3.78%), *Prevotella_7* (18.7 ± 1.95%), *Prevotella_1* (11.01 ± 0.95%), *Ruminococcaceae_UCG-014* (3.31 ± 0.36%), *Succiniclasticum* (3.13 ± 0.81%), *Dialister* (2.36 ± 0.35%), *Rikenellaceae_RC9_gut_group* (1.47 ± 0.21%), *Candidatus_Saccharimonas* (1.16 ± 0.14%), *Shuttleworthia* (1.049 ± 0.087%), and *Ruminococcus_1* (1.00 ± 0.12%). Four genera, namely, *Ruminococcus_gauvreauii_group*, *Butyrivibrio_2*, *Moryella*, and *Ruminiclostridium_6*, were significantly different (*P* < 0.05) after filtering species whose abundance was < 0.1% in all samples. Among the three genera, namely, *Ruminococcus_gauvreauii_group*, *Butyrivibrio_2*, and *Moryella*, the highest abundance was found in the D-A group. In the D-B group, *Ruminiclostridium_6* is the highest abundance (Figure 4).

### Macrogenome and functional profiles of rumen microbiota

**FIGURE 5:**
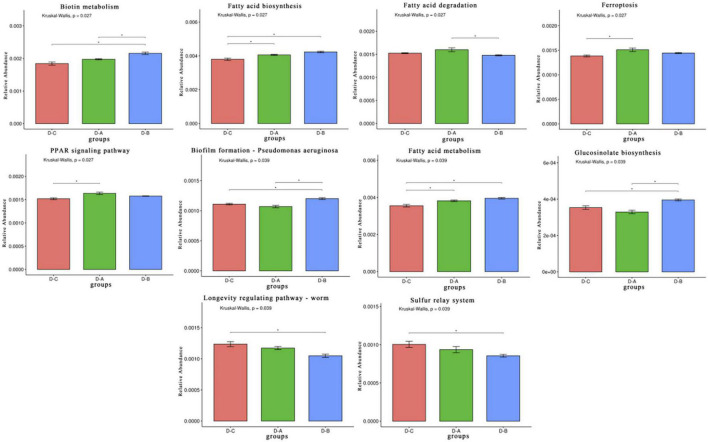
Rumen microbiota functional profiles. Differential Kyoto Encyclopedia of Genes and Genomes (KEGG) pathways were selected. *P < 0.05.

A total of 650,894,398 ± 4,988,354 clean reads were sequenced using the Illumina platform after quality control filtering. The sequences were then assembled to obtain 4,417,583 contigs. After gene annotation of the contigs and obtaining non-redundant gene sets, unigenes were compared with the KEGG database and 408 tertiary pathways were assessed. Of these, ten pathways differed significantly among the three phyla (*P* < 0.05), including five metabolic pathways, two cellular processing pathways, two organismal systems, and one genetic information processing pathway (Figure 5).

### Metabolomics of the rumen fluid

**FIGURE 6:**
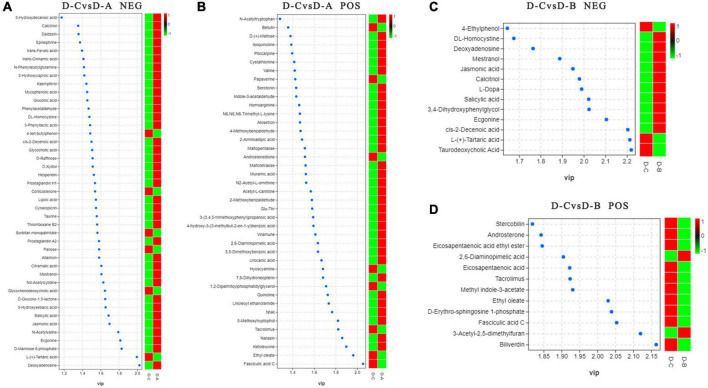
Distinguishing metabolites in the rumen fluid. **(A)** Differential metabolites between D-C and D-A in the neg mode, **(B)** differential metabolites between D-C and D-A in the pos mode, **(C)** differential metabolites between D-C and D-B in the neg mode, and **(D)** differential metabolites between D-C and D-B in the pos mode. VIP > 1, *P* < 0.05.

**FIGURE 7:**
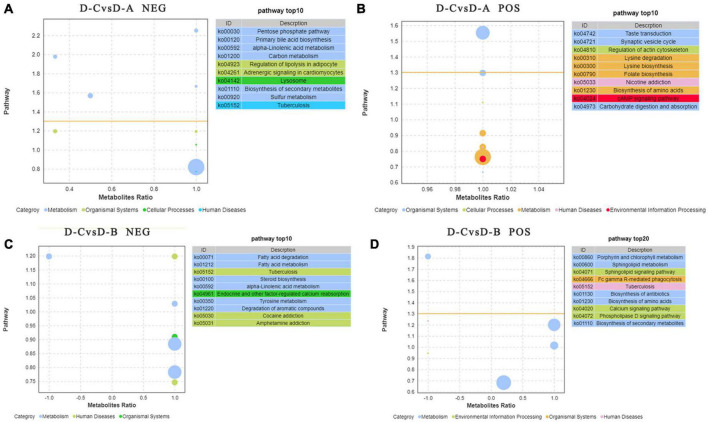
Pathways with significant enrichment in differential metabolites were selected, and bubble maps were drawn using information about upregulation and downregulation. The *X*-axis represents the difference between the number of upregulated and downregulated differential metabolites as a percentage of total differential metabolites. The yellow line represents the threshold of *P* = 0.05. The *Y*-axis represents –log10 (*P*-value). Different colors represent different categories. **(A)** Enrichment difference bubble chart of metabolites between D-C and D-A in the neg mode, **(B)** enrichment difference bubble chart of metabolites between D-C and D-A in the pos mode, **(C)** enrichment difference bubble chart of metabolites between D-C and D-B in the neg mode, and **(D)** enrichment difference bubble chart of metabolites between D-C and D-B in the pos mode.

To further assess the differences in rumen metabolites, the rumen fluid metabolomics were determined. We compared the D-C group with the D-A and D-B groups and identified 41 differential metabolites in the pos mode of the D-C vs. D-A group, 43 in the neg mode, 12 in the D-C vs. D-B pos mode, and 13 in the neg mode (*P* < 0.05, VIP > 1) (Figure 6). According to the differential metabolites, 50 enriched pathways were identified in the pos pattern of the D-C vs. D-A groups. The taste transduction pathway was significantly enriched, including upregulation of the four metabolites, D-phenylalanine, maltose, acetylcholine, and serotonin in the D-A group. In the neg model, 52 enriched pathways were identified, of which four, pentose phosphate, primary bile acid biosynthesis, α-linolenic acid metabolism, and carbon metabolism, were significantly enriched. In the pentose phosphate pathway, gluconic acid and D-glucono-1,5-lactone were upregulated in the D-A group and in the primary bile acid biosynthesis pathway, taurine and glycocholic acid were upregulated, sugarcane deoxycholic acid was downregulated in the D-A group and in the α-linolenic acid metabolism pathway, jasmonic acid was upregulated in the D-A group and in carbon metabolism, and gluconic acid and D-glucono-1,5-lactone were upregulated in the D-A group. In the D-C vs. D-B group, 21 enrichment pathways were found in the pos mode, of which one metabolic pathway, porphyrin, and chlorophyll metabolism were upregulated, and biliverdin and fecal biliverdin were downregulated in the D-B group. Twenty-five enriched pathways were found in the neg model, but none were significantly enriched (Figure 7).

## Discussion

To understand the mechanism by which *S. cerevisiae* cultures alleviate heat stress in dairy cows, the experiment investigated the effects of *S. cerevisiae* cultures on the microbial composition, metagenome, and metabolome of the rumen.

Previous studies indicate that the species diversity and abundance of rumen microorganisms play a critical role in maintaining host physiology (Xu et al., 2021). While Chao1 and ACE indices are primarily used to measure species richness, the Simpson and Shannon indices combine the richness and evenness of a species. Our study found that rumen bacterial abundance and diversity were higher in cows that were fed with the first yeast culture than in the other two groups, although these findings were not statistically significant. This may be due to the increased diversity and abundance of rumen bacteria after the addition of *S. cerevisiae* culture. The environment within the rumen can change to some extent and may affect the bacterial species involved (Ghazanfar et al., 2017). Our results showed that *Bacteroidetes*, *Proteobacteria*, and *Firmicutes* were the dominant phyla in the rumen of all three groups of dairy cows, which supports the findings of prior studies (Xue et al., 2019). The abundance of *Firmicutes* was highest in the D-A group, while the abundance of *Proteobacteria* was lowest in the D-A group; however, these differences were not significant. *Firmicutes* are associated with the degradation of structural polysaccharides (Zened et al., 2013), which may impact the breakdown of cellulose in the rumen. Zou et al. (2019) found that yaks with lower feed conversion efficiency also have a lower relative abundance of *Firmicutes* in the rumen. In contrast, *Proteobacteria* is associated with increased disease risk and an elevated abundance of these bacteria correlates with gut inflammation. *Ruminococcus* and *Firmicutes* are involved in degrading fiber from cellulose-rich feeds (Evans et al., 2011), while *Butyrivibrio* is one of the few genera that can utilize xylan and pectin and degrade hemicellulose, hydrogenate fatty acids, and protein (Bryant and Small, 1956; Orpin et al., 1985; Paillard et al., 2007). *Moryella*, a member of the *Lachnospira* family of *Firmicutes*, can produce short-chain fatty acids through fermentation of dietary polysaccharides (Laffin et al., 2019). Short-chain fatty acids are important substrates for regulating gastrointestinal tract epithelial cells and are able to modulate the inflammatory response (Guo et al., 2020). Our study found a significant increase in the abundance of several genera involved in cellulose catabolism in the D-A group, some of which were also able to produce volatile fatty acids, which decreased the abundance of *Proteobacteria*. These findings indicated that the addition of *S. cerevisiae* culture increased cellulose degradation and reduced inflammation in heat-stressed cows.

The trial assessed the mechanism by which *S. cerevisiae* cultures affect the rumen of heat-stressed cows by metagenome and found ten pathways that differed significantly between the three groups. Fatty acid biosynthesis, fatty acid metabolism, peroxisome proliferator-activated receptor (PPAR) signaling, and ferroptosis and biotin metabolism were all enriched in the treatment groups. Yeast is known as a natural source of B vitamins. According to let’s eat right to keep fit by Adelle Davis, yeast is one of the four foods that contain complete vitamin B. *S. cerevisiae* culture is a product obtained from *S. cerevisiae* by fermentation and concentration, it is also rich in high levels of vitamin B, and studies indicate that vitamin B compounds can increase milk production in dairy cows (Chen et al., 2011). Biotin, a water-soluble B vitamin, is an essential nutrient in the rumen of dairy cows and a required cofactor for five carboxylases. These biotin-dependent carboxylases participate in several metabolic pathways, including fatty acid synthesis and fatty acid oxidation, and biotin or carboxylase deficiency can lead to metabolic dysfunction. Saika et al. (2019) and Lauridsen (2020) found that fatty acids are involved in intestinal mucosal barrier function and oxidative stress and inflammatory responses (Saika et al., 2019; Lauridsen, 2020). By activating fatty acid receptors, they can promote anti-inflammatory and neuronal responses and regulate immune homeostasis (Kimura et al., 2019). The PPAR is also activated by fatty acids and involved in regulating lipid metabolism and inflammation. In addition, PPAR signaling can inactivate NF-κB, promote antioxidant enzyme expression, and reduce the concentration of reactive oxygen species, thus improving antioxidant capacity (Korbecki et al., 2019). Cellulolytic bacteria in the rumen require biotin for growth, and Abel et al. (2001) showed that biotin improves fiber fermentation in the rumen. Thus, we speculated that the addition of *S. cerevisiae* culture increased B vitamin levels in the rumen, which increased biotin metabolism and production, elevated fatty acid synthesis, activated PPAR signaling, and improved the immune response and antioxidant capacity of the cows. The increase in biotin also elevated the abundance of cellulolytic *Firmicutes* in the rumen and improved the digestibility of the feed.

Ferroptosis is an iron-dependent type of programed cell death caused by massive lipid peroxidation-mediated membrane damage. This process is distinct from apoptosis, cellular necrosis, and cellular autophagy (Stockwell et al., 2017; Tang et al., 2019). In most cases, ferroptosis causes an inflammatory response in the body that induces cell death and is harmful to the organism. However, there is evidence that ferroptosis is a double-edged sword and may protect cells from inflammation under certain conditions (Sun et al., 2020). Two groups of cows fed with *S. cerevisiae* cultures in our study were enriched in the ferroptosis pathway, with significant differences between the D-A and control groups, which may be related to increased fatty acid metabolism.

The taste transduction pathway in mammals is not only capable of producing taste but is also expressed in gastrointestinal tract tissues so may play an important role in resisting microbial infections, regulating nutrient absorption, and maintaining internal homeostasis (Yangyu et al., 2017). The receptors for sweetness, freshness, and bitterness are all G protein-coupled, and Minic et al. (2005) found a high degree of homology between the yeast pheromone signaling pathway and mammalian G protein-coupled receptors. G protein-coupled receptors are also expressed in *S. cerevisiae*, which may explain why metabolites are enriched in the taste transduction pathway. The primary breakdown products of cellulose are sucrose and maltose, and according to the sequencing results, the increased abundance of cellulolytic bacteria is associated with elevated maltose levels. G protein-coupled receptors respond to maltose stimulation (Zhao et al., 2003) and release acetylcholine. These results suggest that *S. cerevisiae* cultures may enrich the ruminal taste transduction pathways involved in maintaining internal homeostasis.

The pentose phosphate pathway is the primary pathway for oxidative glucose catabolism in animals and microorganisms and regulates cellular energy supply and cellular respiration, improving metabolic efficiency (Li et al., 2021b). *Ruminococcus* metabolizes pentose *via* the pentose phosphate pathway, so the increased abundance of *Ruminococcus* in this study may account for the upregulation of metabolites associated with the pentose phosphate pathway in the D-A group. The pentose phosphate pathway produces NADPH, which is essential for antioxidant capacity (Patra and Hay, 2014), acting as a hydrogen donor for fatty acid synthesis, participating in fatty acid synthesis, and providing energy to the body (Ohlrogge and Jaworski, 1997). Taurine is a non-essential amino acid that cannot function as a direct energy source (Chesney, 1985) but can provide protection against oxidative stress by increasing superoxide dismutase and glutathione peroxidase activity (Nonaka et al., 2001). Significant enrichment of the pentose phosphate pathway and increased taurine content after the addition of *S. cerevisiae* cultures improved the antioxidant capacity of heat-stressed cows. Glycochenodeoxycholic acid is a hydrophobic bile salt that accumulates in the liver during cholestasis and can induce apoptosis in hepatocytes at certain concentrations. In the D-A group, glycochenodeoxycholic acid production was downregulated, which may have relieved stress on the liver and ensured the health of the organism.

## Conclusion

**FIGURE 8:**
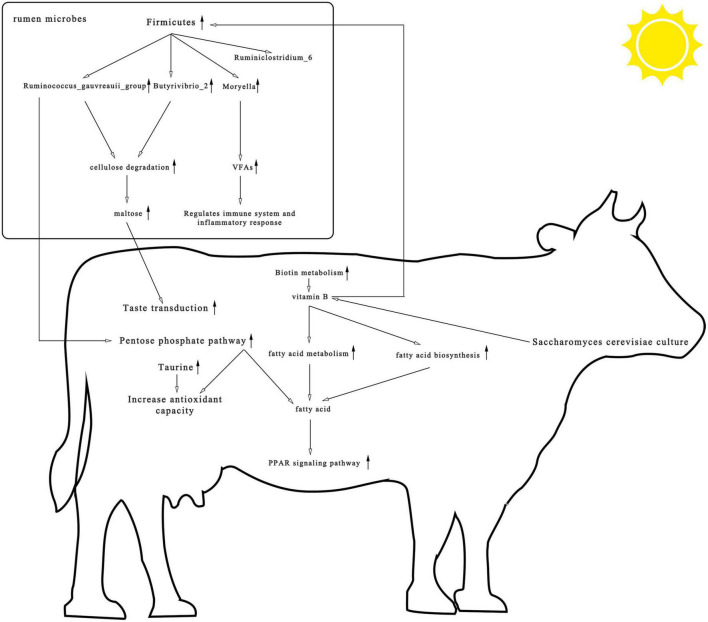
Consolidation of rumen microbial composition, pathways, and differential metabolite results found among heat-stressed cows fed with *Saccharomyces cerevisiae* cultures. The upward arrow represents the increase in rumen microbial composition, pathways, and differential metabolite after the addition of *S. cerevisiae* cultures.

This trial studied the effects of *S. cerevisiae* culture-supplemented diets of heat-stressed cows on rumen microbial composition, metagenomics, and metabolomics and identified a strong relationship between supplementation and vitamin B. As shown in Figure 8, yeast cultures are rich in vitamin B. Supplementation with *S. cerevisiae* culture enriched the rumen in biotin, a water-soluble vitamin B. Biotin can act as a carboxylase cofactor, participating in the synthesis and metabolism of fatty acids, which in turn can activate PPAR and improve the antioxidant capacity and immunity of the organism. In addition, most cellulolytic bacteria require vitamin B for growth, which may account for the increased abundance of cellulolytic bacteria such as *Ruminococcus* and *Butyrivibrio*. These bacteria stimulate cellulose catabolism, upregulate metabolites in the pentose phosphate pathway, increase primary bile acid biosynthesis and taste transduction pathway signaling, and positively impact the health of cows. Our findings indicate that *S. cerevisiae* cultures enriched with vitamin B provide protection against heat stress and should be considered as an anti-heat stress feed additive in production.

## Data availability statement

The datasets presented in this study can be found in online repositories. The names of the repository/repositories and accession number(s) can be found at: https://www.ncbi.nlm.nih.gov/, PRJNA834564.

## Ethics statement

The animal study was reviewed and approved by Ethical Commission of Shandong Agricultural University.

## Author contributions

DD, PC, CW, and ZH conceived the project and designed the protocol. DD and WJ performed the experiments. DD, LF, and YZ wrote the manuscript. All authors read and approved the final manuscript.

## Conflict of interest

PC was employed by Beijing Enhalor International Tech Co., Ltd. The remaining authors declare that the research was conducted in the absence of any commercial or financial relationships that could be construed as a potential conflict of interest.

## Publisher’s note

All claims expressed in this article are solely those of the authors and do not necessarily represent those of their affiliated organizations, or those of the publisher, the editors and the reviewers. Any product that may be evaluated in this article, or claim that may be made by its manufacturer, is not guaranteed or endorsed by the publisher.

## Footnotes

1
https://www.omicshare.com/

2
https://www.mzcloud.org/

3
https://www.omicsmart.com/index.php
