# Supplementary figures and images for: Effect of Saccharomyces cerevisiae culture mitigates heat stress-related dame in dairy cows by multi-omics
Source: Front Microbiol. 2022 Jul 15;13:935004. doi: 10.3389/fmicb.2022.935004 (PMC9335076; doi:10.3389/fmicb.2022.935004)

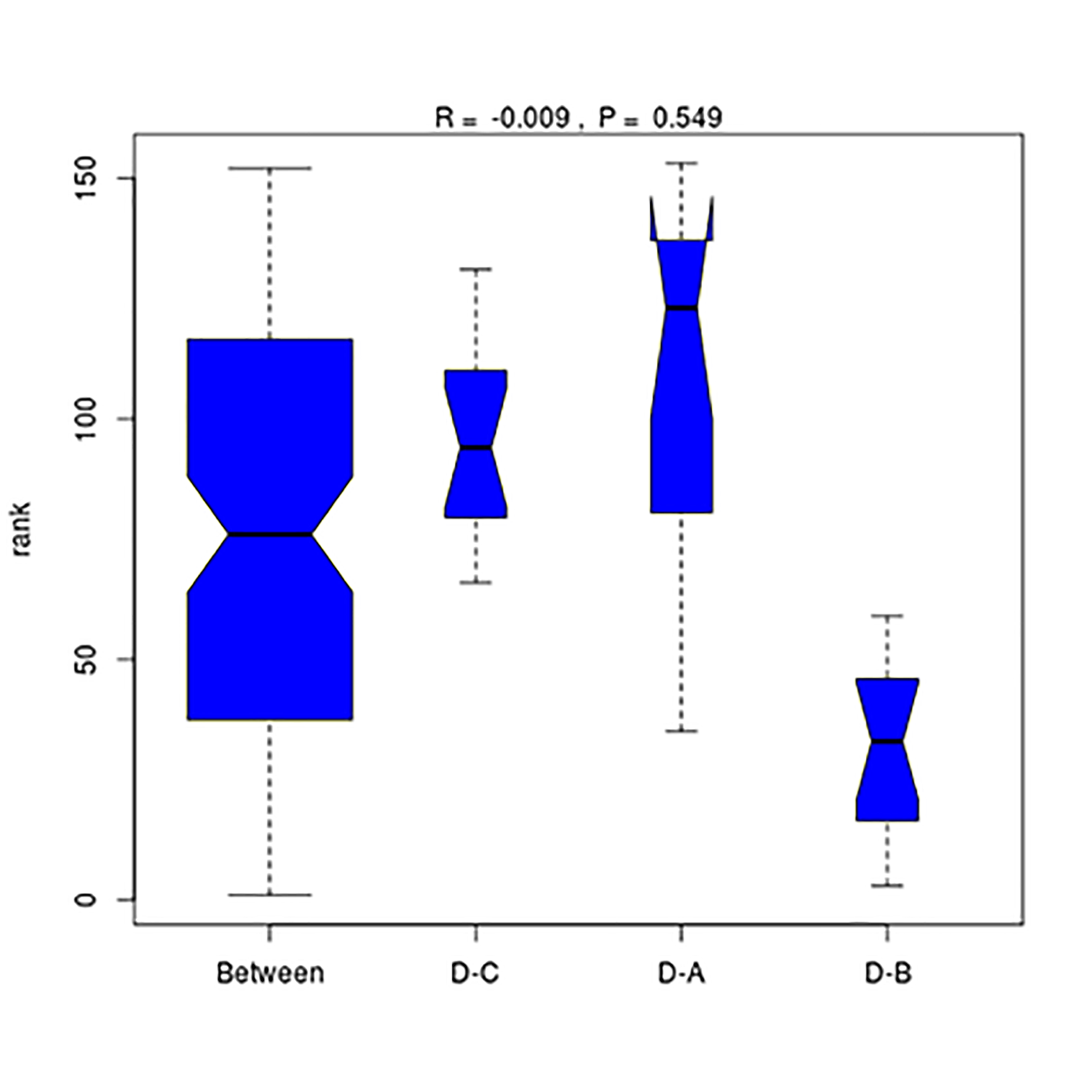

Supplement: Supplementary file 1 [file Image_1.JPEG]
